# Synchronous atrioventricular sequential pacing utilizing conventional and leadless pacemakers in an elderly patient: a case report

**DOI:** 10.1093/ehjcr/ytac474

**Published:** 2022-12-12

**Authors:** Neil Bodagh, Kevin Cheng, William Eysenck, Tom Wong

**Affiliations:** King's College London, St Thomas' Hospital, Westminster Bridge Road, London, SE1 7EH, UK; Royal Brompton Hospital, Sydney Street, London, SW3 6NP, UK; Royal Brompton Hospital, Sydney Street, London, SW3 6NP, UK; Royal Brompton Hospital, Sydney Street, London, SW3 6NP, UK

**Keywords:** Case report, Leadless pacemaker, Bradycardia, Lead failure

## Abstract

**Background:**

Leadless pacemakers present a viable alternative to conventional transvenous devices to mitigate the risk of lead- and/or pocket-related complications. In elderly patients who have encountered ventricular lead failures with transvenous pacemakers, this option may enable the delivery of atrioventricular synchronous pacing therapy, while obviating the requirement for lead extraction and lead-based pacemaker re-implantation.

**Case summary:**

This case report describes the successful implantation of a leadless pacemaker in a 90-year-old who had undergone two dual-chamber permanent pacemaker implantation procedures with a failure of three of four previously implanted leads. Atrioventricular synchronous pacing was achieved, as the leadless device was able to track the atrial-paced rhythm from the pre-existing right-sided device.

**Discussion:**

In elderly patients who have encountered issues with transvenous pacemakers, alternative approaches should be considered to mitigate the risk of future complications. Leadless pacemakers may offer a low-risk solution, enabling the delivery of atrioventricular synchronous pacing therapy in such patient groups. Future studies should be designed to delineate whether these devices could be utilized as a first-line approach in certain situations.

**Learning points**

- Leadless pacemakers are feasible treatment options in pacing-dependent patients who have encountered complications with conventional lead-based devices.
- Atrioventricular synchronous pacing can be achieved with leadless pacemakers via pre-existing devices with functioning atrial leads.
- Further studies are warranted to ascertain whether leadless pacemakers could be considered as a first-line approach in elderly patients with multiple comorbidities to mitigate complication risks and limit the requirement for repeat procedures.

## Introduction

Leadless pacemakers offer the ability to avert complications associated with lead-based pacing related to the generator and leads.^1^ Previous leadless ventricular devices were unable to achieve atrioventricular sequential pacing. While dual-chamber leadless pacemakers are in development, these devices are currently under relicensing investigation and unavailable for routine clinical use.^2^ Newer leadless ventricular pacemakers can achieve atrioventricular synchrony through accelerometer-based atrial sensing.^3^ We describe a case of successful leadless pacemaker implantation in a 90-year-old with a history of multiple lead failures. The leadless pacemaker was able to track the atrial-paced rhythm from a pre-existing device to enable atrioventricular synchronous pacing.

## Timeline

**Table:** 

Eight years prior	Left-sided dual-chamber pacemaker inserted for complete heart block
Three years prior	Right-sided dual-chamber pacemaker insertion and explantation of the left-sided device due to lead failure
Day 1	Presentation to hospital with new-onset intermittent dizziness
Day 1	Temporary pacing wire via the right femoral vein
Day 1	Temporary externalized VVIR pacemaker implant
Day 7	Leadless pacemaker implant

## Case presentation

A 90-year-old gentleman presented to his local hospital with new-onset intermittent dizziness. He had a past medical history of coronary artery bypass surgery (over two decades prior), hypertension, chronic obstructive pulmonary disease, and hyperthyroidism. He had a previous left-sided dual-chamber pacemaker inserted 8 years prior for complete heart block. However, due to a failure of insulation and increased noise detection from both the ventricular and the atrial leads with the inhibition of pacing (causing symptoms of dizziness), he had a further right-sided dual-chamber pacemaker inserted via the subclavian vein 3 years prior. This was accompanied by explantation of the left-sided box and burial of the leads. Therefore, at the time of his presentation, he had four pacing leads *in situ*. Otherwise, he was a non-smoker, did not drink alcohol, lived alone, and was independent with activities of daily living. His physical examination was unremarkable except for a soft ejection systolic murmur consistent with a known diagnosis of mild aortic stenosis.

**Figure 1:**
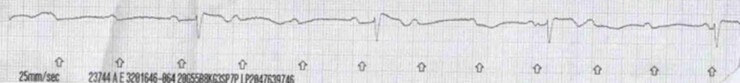
Electrocardiogram rhythm strip exhibiting a complete heart block.

**Figure 2:**
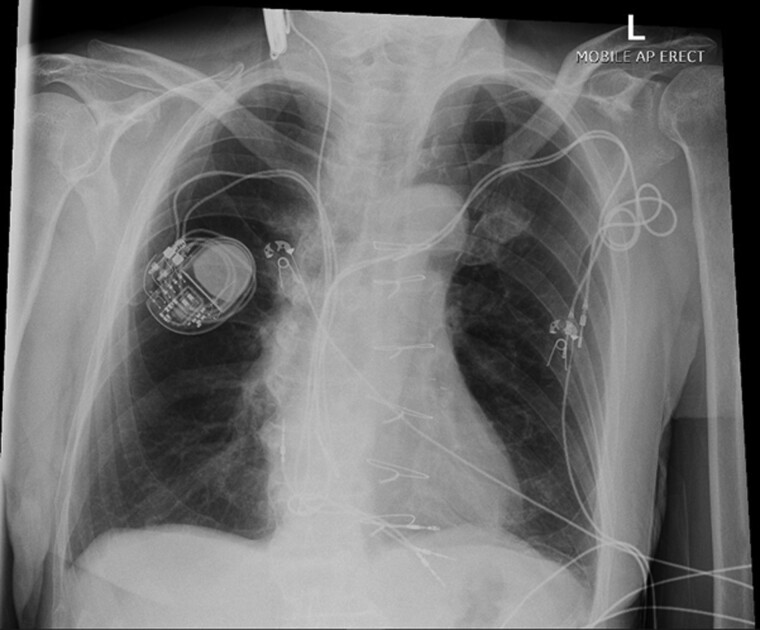
Chest X-ray following external pacemaker implantation demonstrating the presence of five right-sided leads (two leads from the left-sided device, two leads from the right-sided device, and one lead from the external pacemaker).

The patient’s electrocardiogram rhythm strip demonstrated the presence of a complete heart block (*Figure 1*). An urgent pacing check revealed right ventricular lead failure with intermittent and subsequently complete loss of right ventricular capture when the patient moved his arms. An urgent temporary pacing wire was inserted through the right femoral vein ensuring stabilization before transfer to his local cardiac centre. An urgent temporary externalized VVIR pacemaker implant was performed, and the femoral temporary wire was removed (*Figure 2*).

Blood tests were unremarkable with normal electrolytes [sodium 137 mmol/L (reference range: 133–146 mmol/L); potassium 4.5 mmol/L (reference range: 3.5–5.3 mmol/L); corrected calcium 2.51 mmol/L (reference range: 2.20–2.60 mmol/L); phosphate 1.02 mmol/L (reference range: 0.80–1.50 mmol/L); magnesium 0.91 mmol/L (reference range: 0.7–1.0 mmol/L)]. A transthoracic echocardiogram showed a normal-sized left ventricle with a basal septal bulge and borderline-reduced global systolic function (ejection fraction 55%). There was mild aortic stenosis and tricuspid regurgitation and no other significant valvular pathology.

Given the failure of three of four previous implanted leads in this patient, several potential strategies for a permanent system were discussed. This included the implantation of another right-sided dual-chamber pacemaker (a venogram confirmed patency of the right-sided axillary and subclavian veins) with potential laser lead extraction. Considering the patient’s previous history of lead failures and the difficulty of further lead insertion and risks of lead extraction, a leadless pacemaker was considered the best option in this patient.

**Figure 3:**
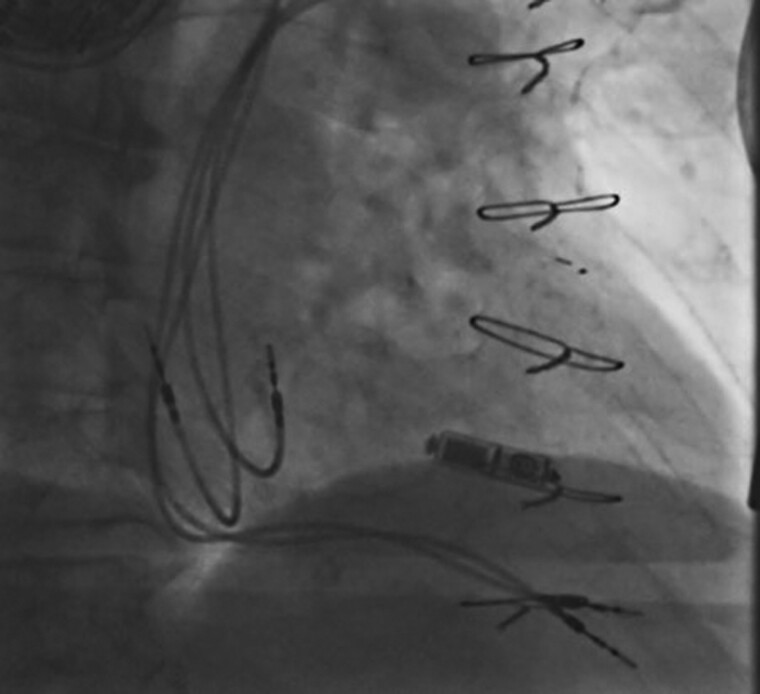
Fluoroscopic view demonstrating the final position of the leadless pacemaker in the right ventricular septum.

**Figure 4:**
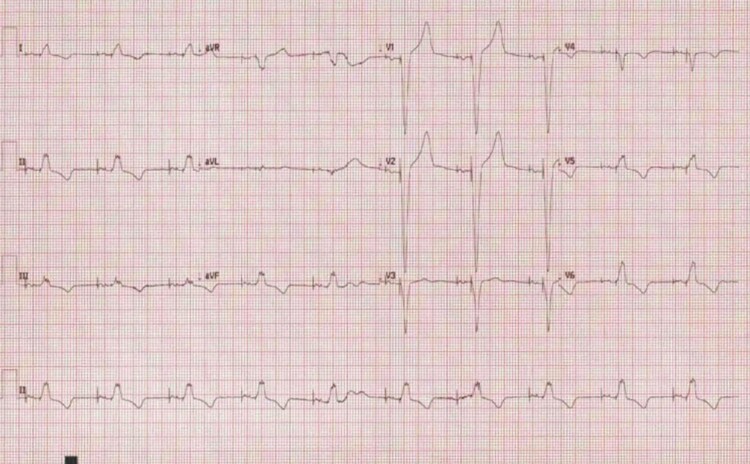
Electrocardiogram post leadless pacemaker insertion demonstrating atrioventricular sequential pacing, as the leadless pacemaker was able to track the atrial-paced rhythm from a pre-existing device.

The patient underwent implantation of a Micra atrioventricular leadless pacemaker under local anaesthetic and sedation (1 mg midazolam, 1 mg morphine, and 10 mg metoclopramide). Through right femoral venous access (7F sheath inserted and upsized to 16F), the delivery system was advanced, and the Micra atrioventricular leadless device was positioned in the septal position with good initial measurements (*Figure 3*). The device was programmed to VDI at 50 b.p.m. The total duration of the procedure was 48 min with 15 mL of Visipaque contrast used and a radiation dosage of 502.9 mGy/cm^2^. Post-procedural pacing checks showed sinus rhythm with no intrinsic ventricular rhythm over VVI at 30 b.p.m. The existing functional atrial lead from the right-sided device had a satisfactory threshold (0.75 V at 0.4 ms), impedance (418 Ω), and p-wave amplitude (3.3 mV). The Micra atrioventricular device sensing was satisfactory, tracking the atrial rhythm at 60 b.p.m. (AAIR pacing from the permanent right-sided system; *Figure 4*). Frequencies of AM-VP (atrial mechanical event-ventricular pacing reflecting atrioventricular synchrony during ventricular pacing) and VP (ventricular pacing) were 93.3% and 6.5%, respectively. There were no immediate complications, and the patient was discharged home the following day.

**Figure 5:**
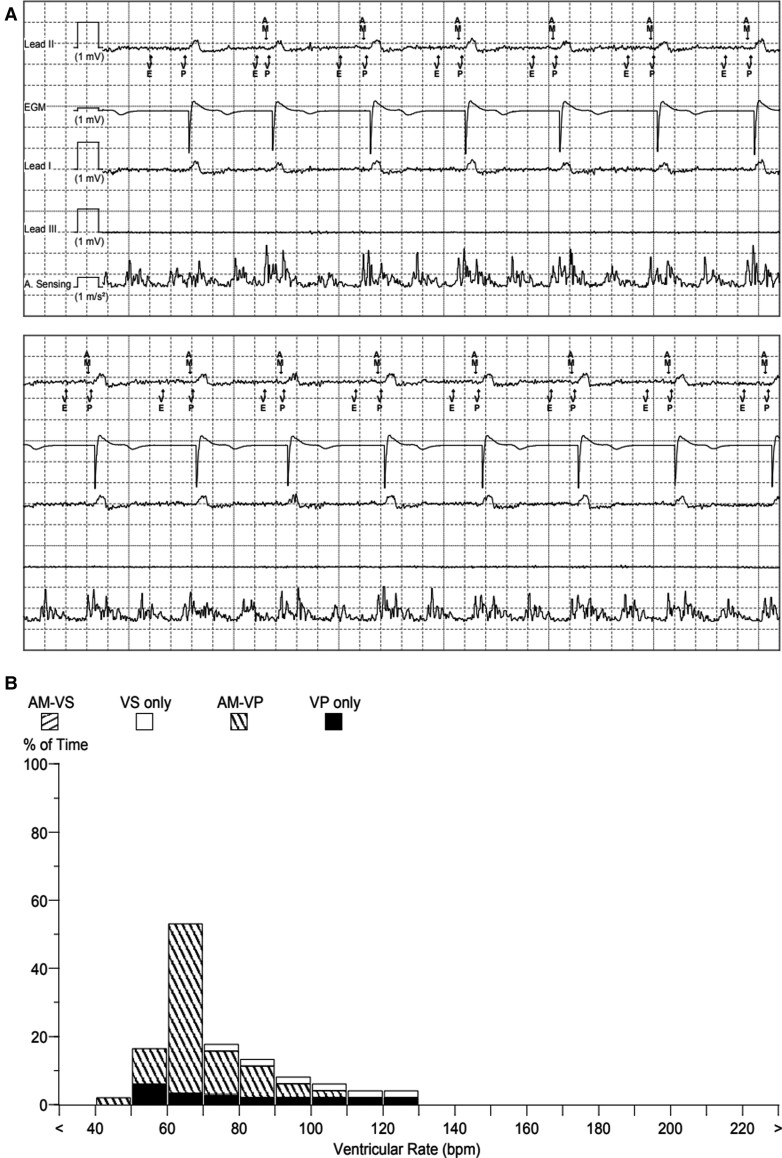
(*A*) Pacing system analysis demonstrating atrial mechanical sensing and atrioventricular synchronous pacing. (*B*) Micra atrioventricular leadless pacemaker histogram.

At 6 weeks of follow-up, the pacing check revealed a presenting rhythm of AP (from the transvenous pacemaker) and VP (from the Micra device) with appropriate atrioventricular tracking (*Figure 5A*). The frequencies of AM-VP and VP were 86.9% and 12.8%, respectively (*Figure 5B*). The patient’s underlying rhythm was a complete heart block.

## Discussion

Our case highlights the feasibility of leadless pacemaker implantation in an elderly patient with a history of multiple lead failures. While transvenous pacemakers are well-established treatment modalities for bradyarrhythmia, lead- and/or pocket-related complications present major limitations to their use.^4^ The leadless pacemaker has emerged as a solution to reduce complications associated with transvenous pacing.

The patient described in the current case report had undergone two permanent pacemaker implants with a failure of three of four previously implanted leads. The subsequent femoral temporary wire and external VVIR pacemaker implant resulted in a total of six right-sided pacemaker lead at one stage. Lead-related complications are unfortunately common following transvenous pacemaker implantation,^5^ with one meta-analysis reporting a mean incidence of lead failure of 1.78%.^6^ Risk factors for lead complications include venous access technique,^7^ operator inexperience,^8^ and implantation in low-volume centres.^5^ In this study, details regarding venous access for the left-sided device were unavailable. However, it is notable that subclavian access was utilized for the right-sided device and this method has been associated with a higher rate of lead-related complications.^7^ Considering the patient’s history of lead failures, a multidisciplinary team discussion was necessary to determine the optimal treatment strategy.

Dual-chamber pacemaker implantation with laser lead extraction was considered. However, this approach presented risks associated with both a third permanent pacemaker implant procedure and lead extraction. A further permanent pacemaker implant procedure with leads entering the heart via the superior vena cava may have heightened the risk of superior vena cava obstruction.^9^ Furthermore, transvenous lead extraction is a technically challenging procedure and an increased number of leads extracted has been associated with a higher complication rate.^10^ These issues emphasized the requirement for an alternative approach.

Our case report demonstrates that leadless pacemaker implantation can be performed uneventfully in a 90-year-old patient with multiple lead failures and co-morbidities mitigating the risks associated with transvenous pacemaker implantation and lead extraction. This is in keeping with real-world data demonstrating high degrees of safety of leadless pacemaker implantation across all patient age groups.^11^ Indeed, the procedure may result in shorter procedure times in elderly patients.^12^ Future studies could attempt to ascertain whether leadless pacemaker implantation may offer benefit as a first-line pacing approach.

Previous leadless pacing devices provided ventricular-only pacing limiting candidate selection to patients with an indication for a VVIR pacemaker or those with a sufficient contraindication to transvenous lead placement to rationalize ventricular-only pacing.^13^ However, the Micra Atrial Tracking Using a Ventricular Accelerometer 2 (MARVEL 2) study has demonstrated the feasibility of accelerometer-based atrial sensing with leadless ventricular pacemakers, thereby enabling atrioventricular synchrony.^3^ The accelerometer can detect four mechanical signals corresponding to various stages of the cardiac cycle.^14^ The A1 and A2 signals correspond to tricuspid/mitral and aortic/pulmonary valve closure, respectively. A3 indicates passive ventricular filling, whilst A4 corresponds to atrial contraction. Thresholds are set to avoid A3 ventricular sensing and ensure atrial A4 sensing.

In this study, the leadless pacemaker was able to track the atrial-paced rhythm from the permanent right-sided system. The ability of leadless pacemakers to track atrial-paced rhythms from pre-existing devices has previously been reported.^15,16^ Our study confirms this to be feasible in an elderly patient with multiple lead failures. Whilst leadless atrial pacemakers are in development,^17,18^ this finding demonstrates the potential for current leadless ventricular pacemakers to enable atrioventricular synchrony with or without pre-existing devices. This has the potential to further expand the utility of leadless pacemakers. In elderly patients, it has previously been reported that the use of DDD pacing is associated with reduced mortality rates in comparison with VVI pacing.^19^ This iterates the importance of atrioventricular sequential pacing in this patient cohort.

## Conclusion

Our case demonstrates how a leadless pacemaker can be utilized to achieve atrioventricular synchronous pacing in a 90-year-old patient with a pre-existing device and a history of multiple lead failures. Leadless pacemakers offer the potential to mitigate risks associated with transvenous pacemaker implantation. Further studies could be performed to ascertain whether lead failure could emerge as an indication for leadless pacing and identify potential instances where these devices could be considered as a first-line approach.

## Supplementary Material

ytac474_Supplementary_Data

## Lead author biography

Neil Bodagh studied Medicine at the University of Manchester. He is now a Cardiology Clinical Research Fellow in London.
