# Supplementary material for: Synchronous atrioventricular sequential pacing utilizing conventional and leadless pacemakers in an elderly patient: a case report
Source: Eur Heart J Case Rep. 2022 Dec 12;7(1):ytac474. doi: 10.1093/ehjcr/ytac474 (PMC9851417; doi:10.1093/ehjcr/ytac474)
Supplement: ytac474_Supplementary_Data [file ytac474_supplementary_data.pptx]

## Slide 1
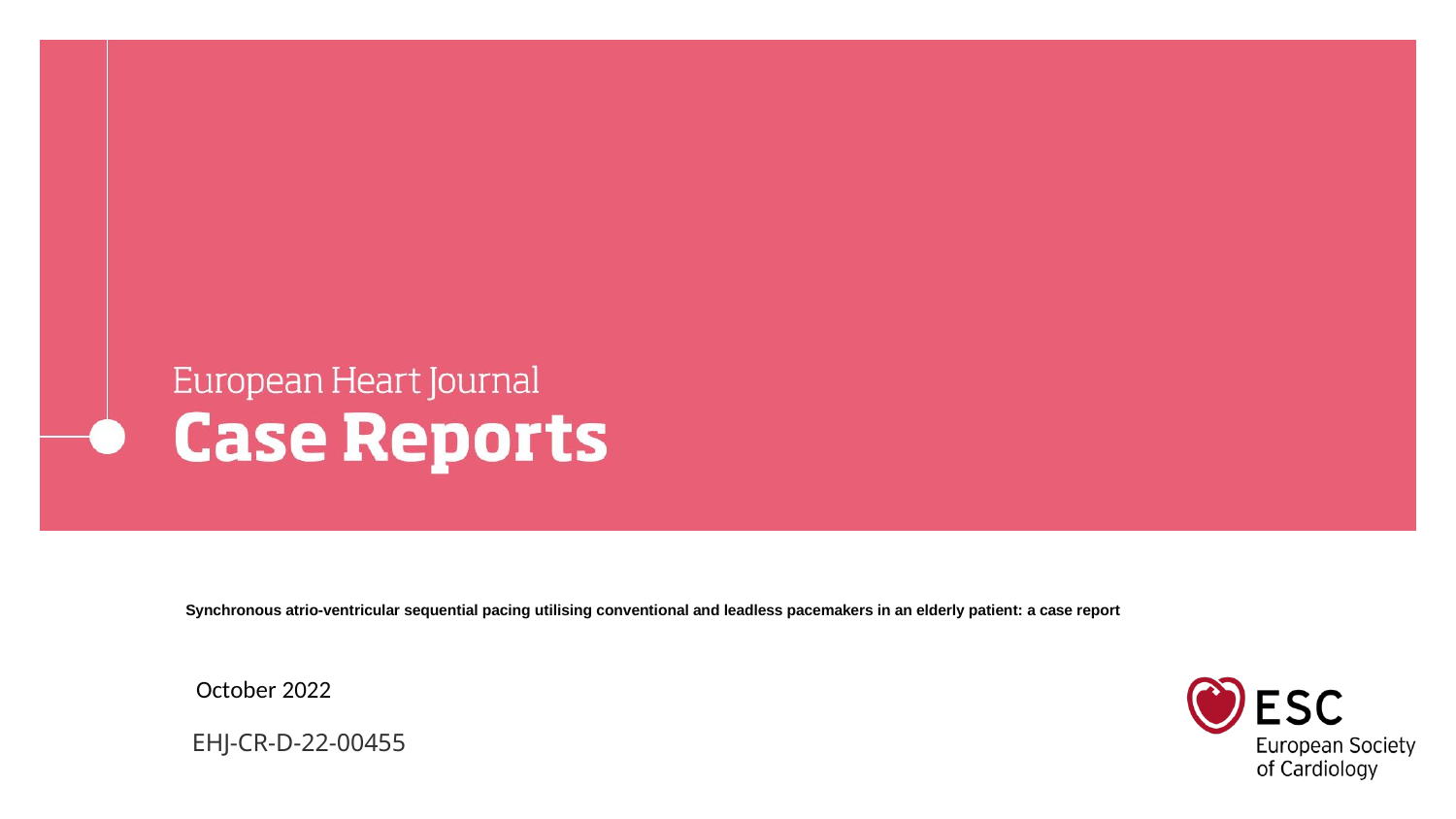

# Synchronous atrio-ventricular sequential pacing utilising conventional and leadless pacemakers in an elderly patient: a case report
October 2022
EHJ-CR-D-22-00455

## Slide 2
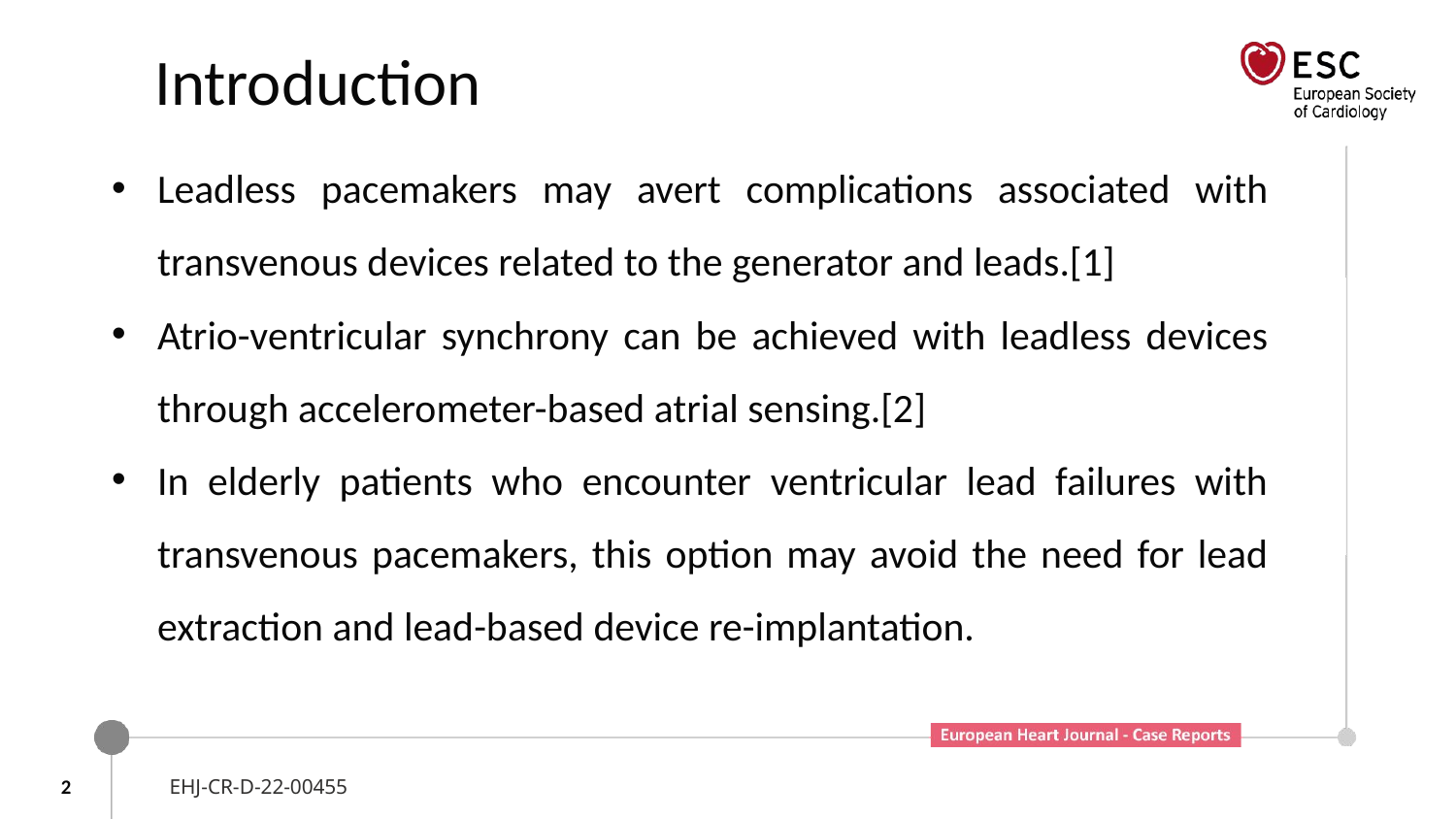

# Introduction
Leadless pacemakers may avert complications associated with transvenous devices related to the generator and leads.[1]
Atrio-ventricular synchrony can be achieved with leadless devices through accelerometer-based atrial sensing.[2]
In elderly patients who encounter ventricular lead failures with transvenous pacemakers, this option may avoid the need for lead extraction and lead-based device re-implantation.
2
EHJ-CR-D-22-00455

## Slide 3
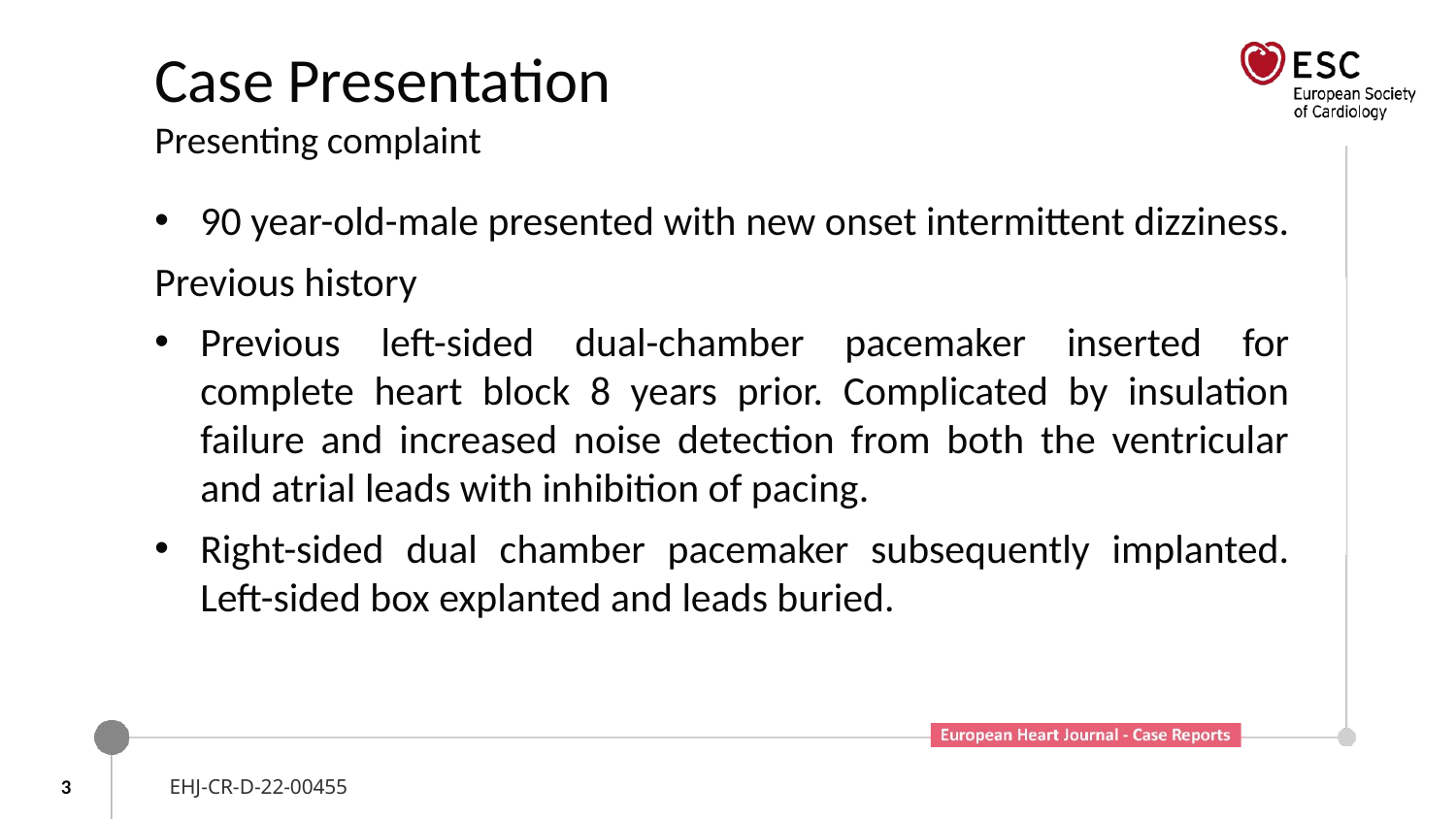

# Case Presentation Presenting complaint
90 year-old-male presented with new onset intermittent dizziness.
Previous history
Previous left-sided dual-chamber pacemaker inserted for complete heart block 8 years prior. Complicated by insulation failure and increased noise detection from both the ventricular and atrial leads with inhibition of pacing.
Right-sided dual chamber pacemaker subsequently implanted. Left-sided box explanted and leads buried.
3
EHJ-CR-D-22-00455

## Slide 4
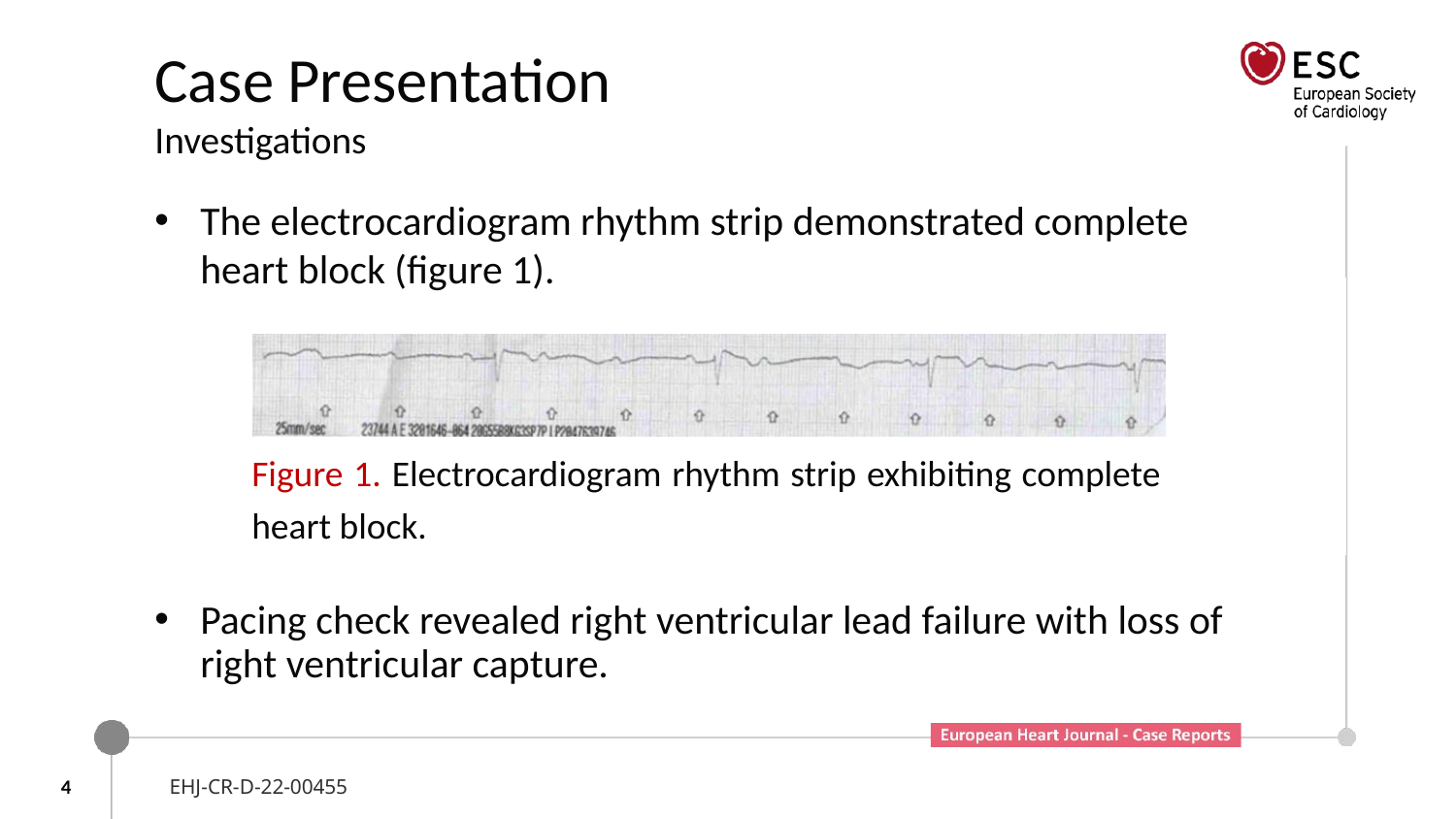

# Case Presentation Investigations
The electrocardiogram rhythm strip demonstrated complete heart block (figure 1).
Figure 1. Electrocardiogram rhythm strip exhibiting complete heart block.
Pacing check revealed right ventricular lead failure with loss of right ventricular capture.
4
EHJ-CR-D-22-00455

## Slide 5
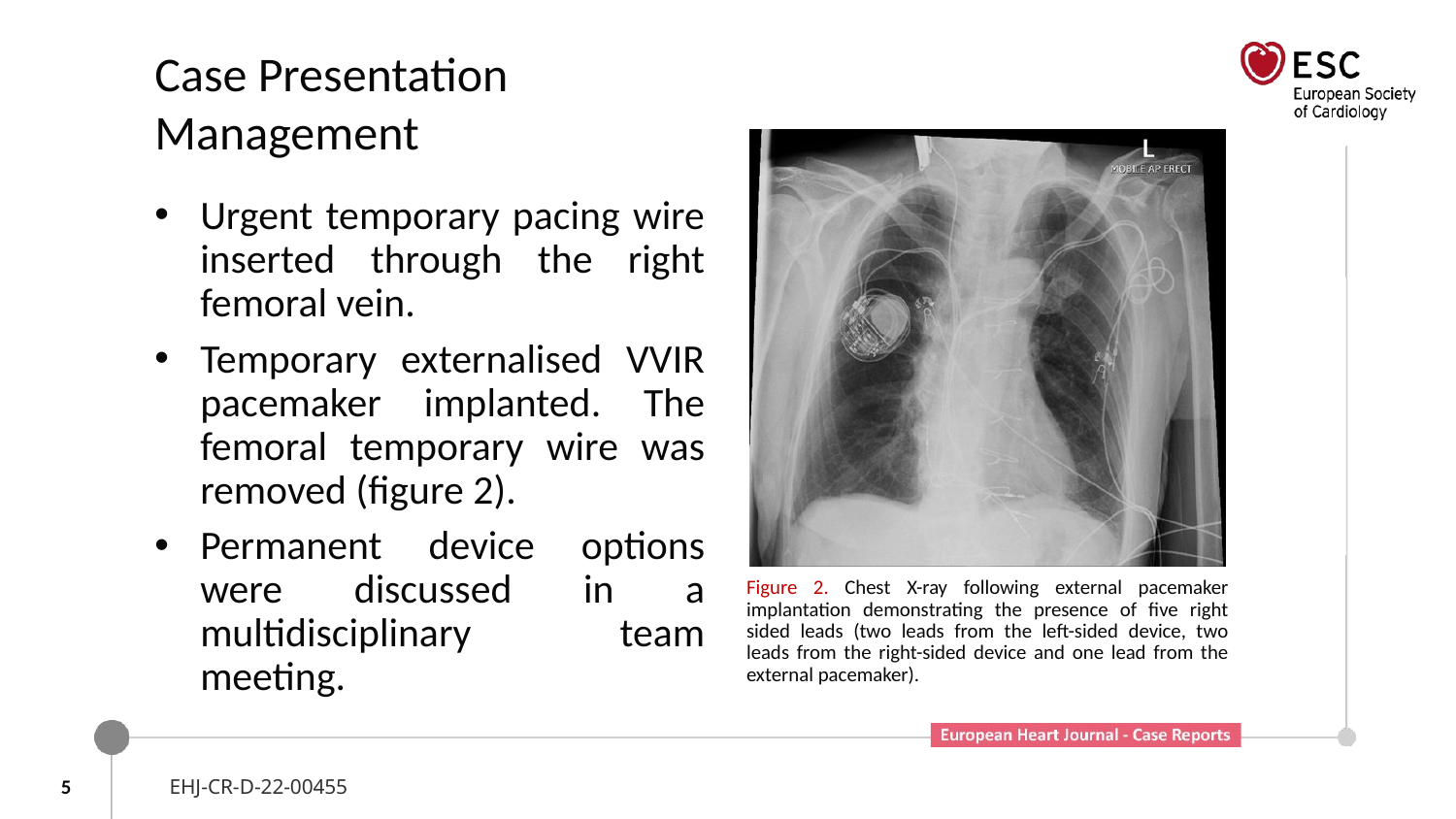

# Case Presentation Management
Urgent temporary pacing wire inserted through the right femoral vein.
Temporary externalised VVIR pacemaker implanted. The femoral temporary wire was removed (figure 2).
Permanent device options were discussed in a multidisciplinary team meeting.
Figure 2. Chest X-ray following external pacemaker implantation demonstrating the presence of five right sided leads (two leads from the left-sided device, two leads from the right-sided device and one lead from the external pacemaker).
5
EHJ-CR-D-22-00455

## Slide 6
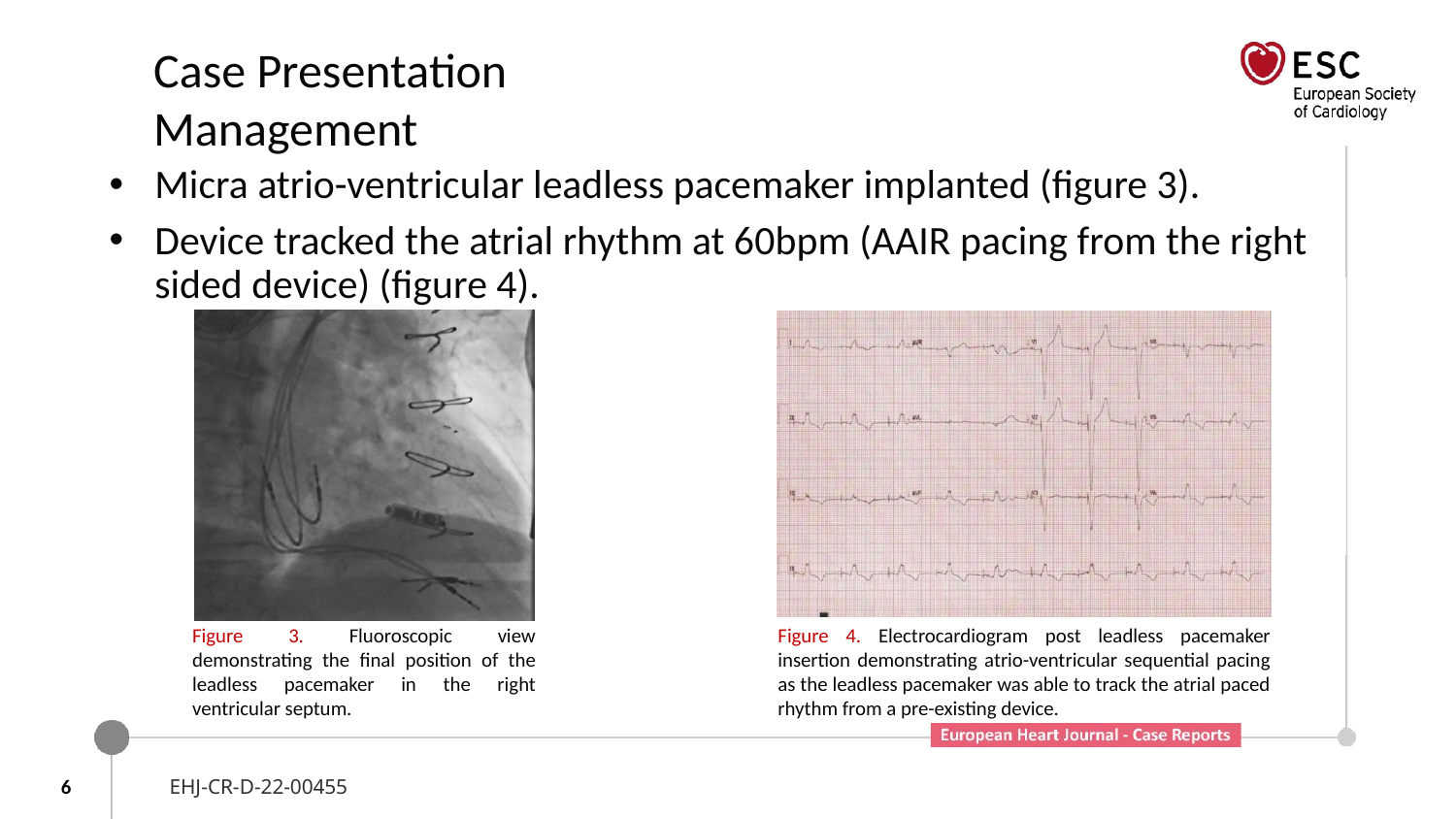

# Case Presentation Management
Micra atrio-ventricular leadless pacemaker implanted (figure 3).
Device tracked the atrial rhythm at 60bpm (AAIR pacing from the right sided device) (figure 4).
Figure 3. Fluoroscopic view demonstrating the final position of the leadless pacemaker in the right ventricular septum.
Figure 4. Electrocardiogram post leadless pacemaker insertion demonstrating atrio-ventricular sequential pacing as the leadless pacemaker was able to track the atrial paced rhythm from a pre-existing device.
6
EHJ-CR-D-22-00455

## Slide 7
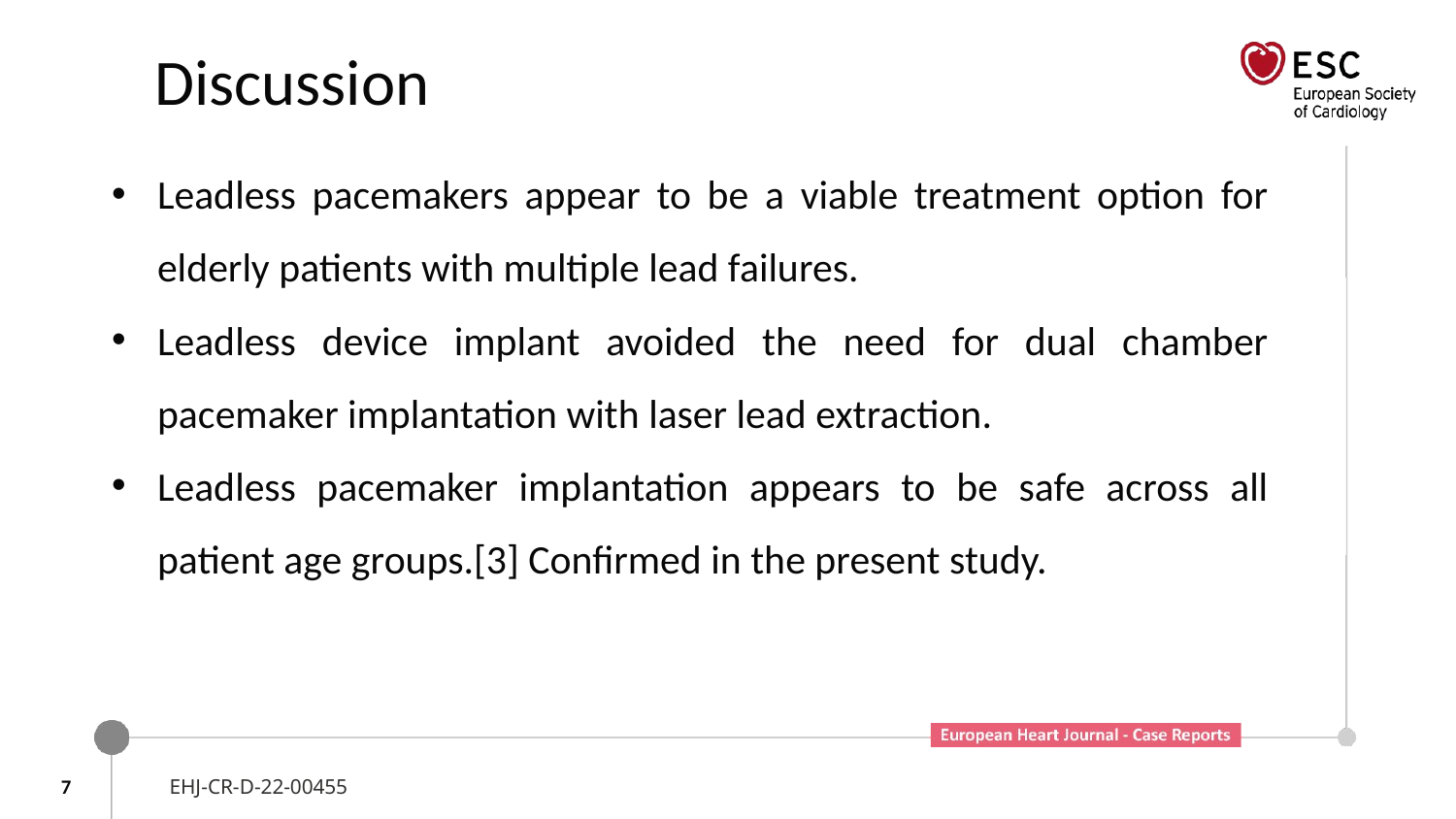

# Discussion
Leadless pacemakers appear to be a viable treatment option for elderly patients with multiple lead failures.
Leadless device implant avoided the need for dual chamber pacemaker implantation with laser lead extraction.
Leadless pacemaker implantation appears to be safe across all patient age groups.[3] Confirmed in the present study.
7
EHJ-CR-D-22-00455

## Slide 8
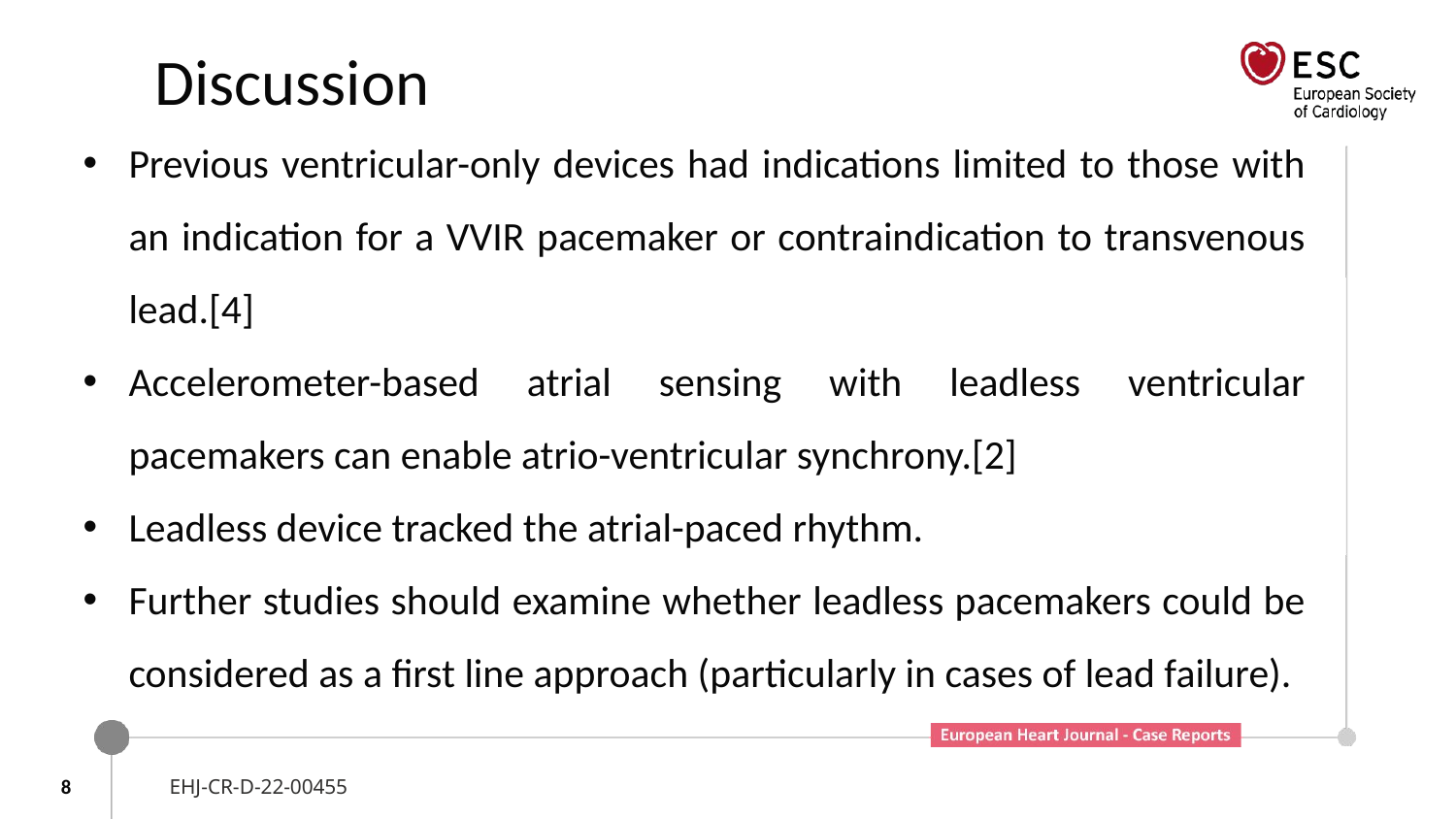

# Discussion
Previous ventricular-only devices had indications limited to those with an indication for a VVIR pacemaker or contraindication to transvenous lead.[4]
Accelerometer-based atrial sensing with leadless ventricular pacemakers can enable atrio-ventricular synchrony.[2]
Leadless device tracked the atrial-paced rhythm.
Further studies should examine whether leadless pacemakers could be considered as a first line approach (particularly in cases of lead failure).
8
EHJ-CR-D-22-00455

## Slide 9
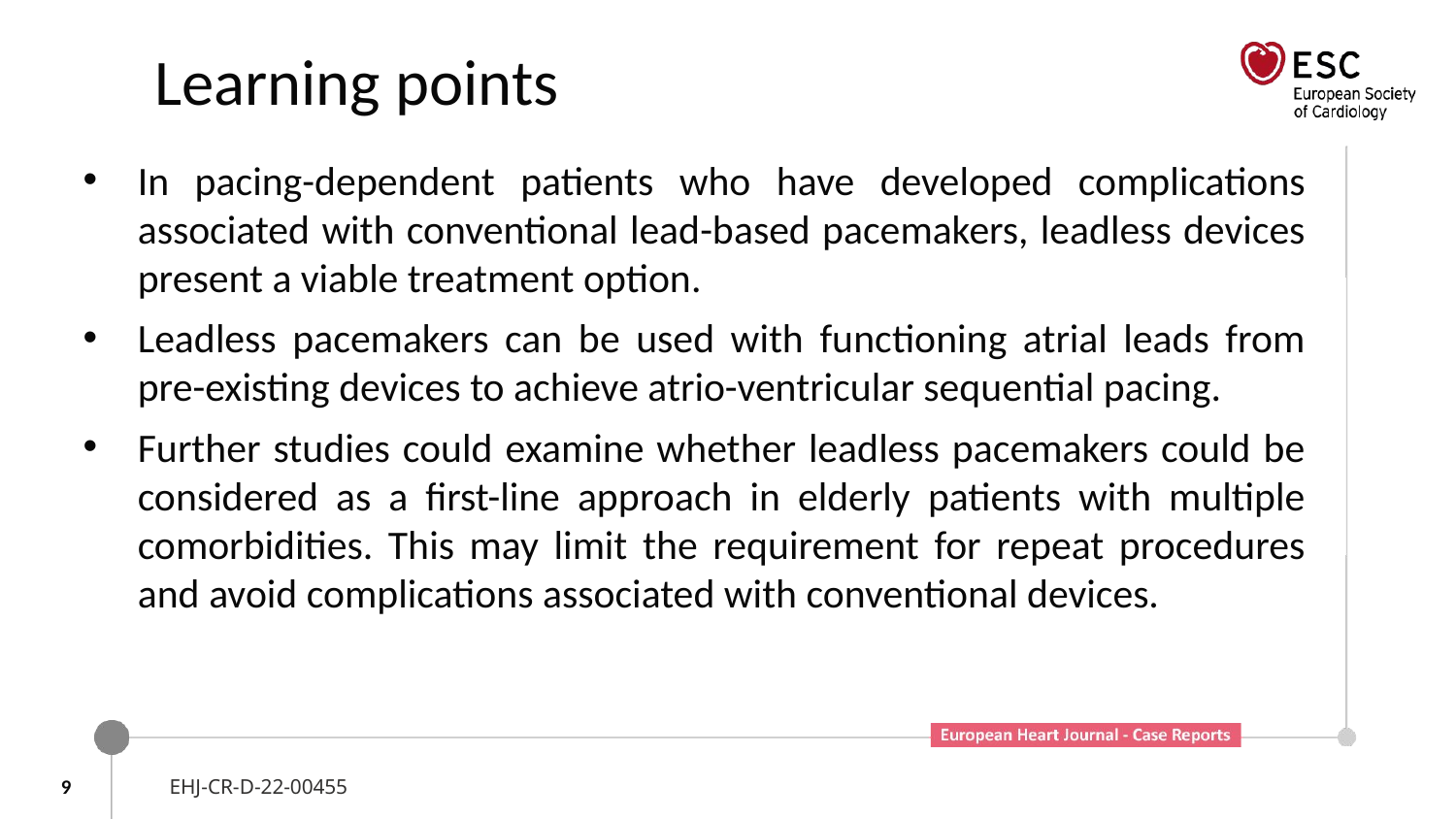

# Learning points
In pacing-dependent patients who have developed complications associated with conventional lead-based pacemakers, leadless devices present a viable treatment option.
Leadless pacemakers can be used with functioning atrial leads from pre-existing devices to achieve atrio-ventricular sequential pacing.
Further studies could examine whether leadless pacemakers could be considered as a first-line approach in elderly patients with multiple comorbidities. This may limit the requirement for repeat procedures and avoid complications associated with conventional devices.
9
EHJ-CR-D-22-00455

## Slide 10
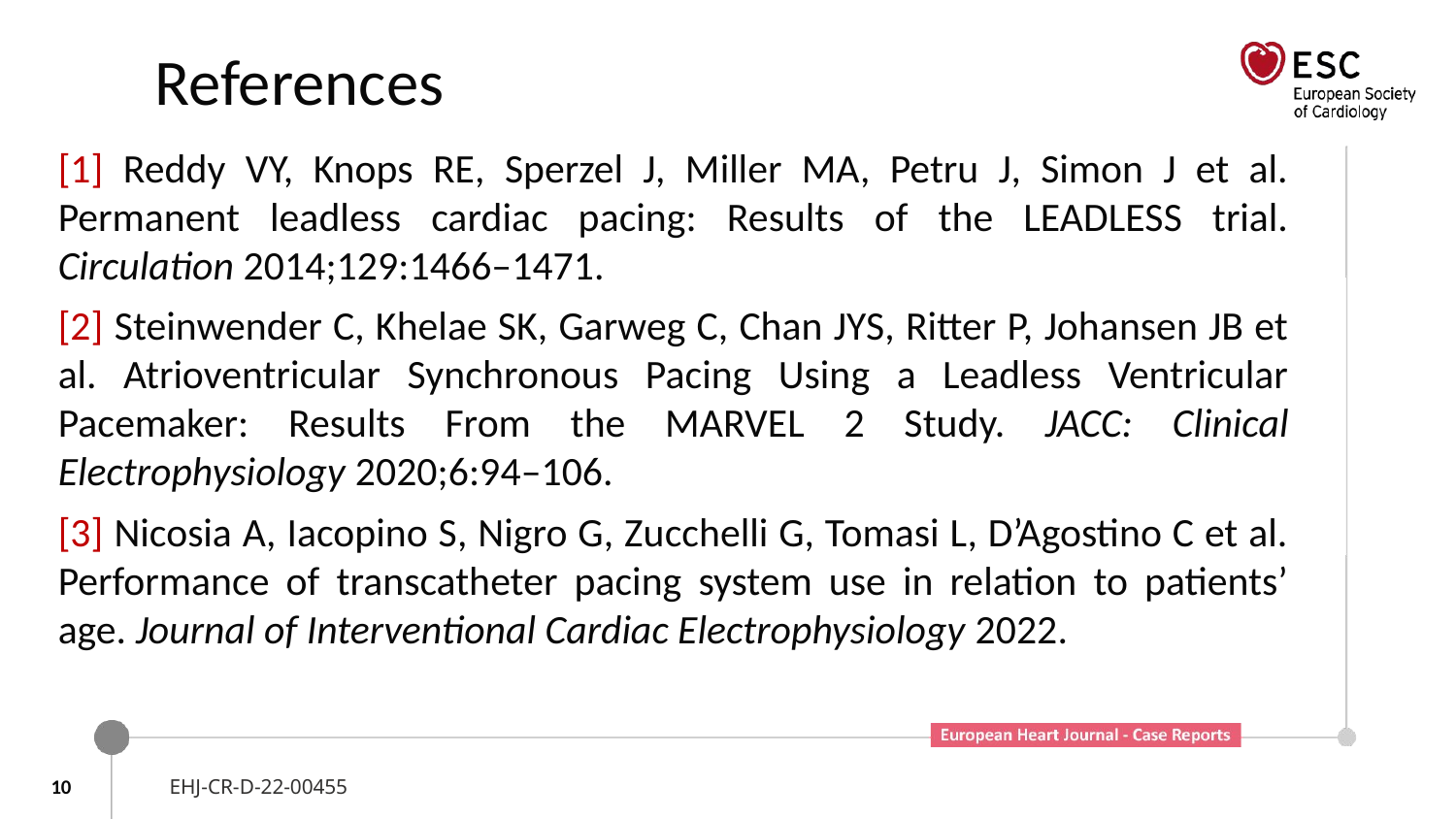

# References
[1] Reddy VY, Knops RE, Sperzel J, Miller MA, Petru J, Simon J et al. Permanent leadless cardiac pacing: Results of the LEADLESS trial. Circulation 2014;129:1466–1471.
[2] Steinwender C, Khelae SK, Garweg C, Chan JYS, Ritter P, Johansen JB et al. Atrioventricular Synchronous Pacing Using a Leadless Ventricular Pacemaker: Results From the MARVEL 2 Study. JACC: Clinical Electrophysiology 2020;6:94–106.
[3] Nicosia A, Iacopino S, Nigro G, Zucchelli G, Tomasi L, D’Agostino C et al. Performance of transcatheter pacing system use in relation to patients’ age. Journal of Interventional Cardiac Electrophysiology 2022.
10
EHJ-CR-D-22-00455

## Slide 11
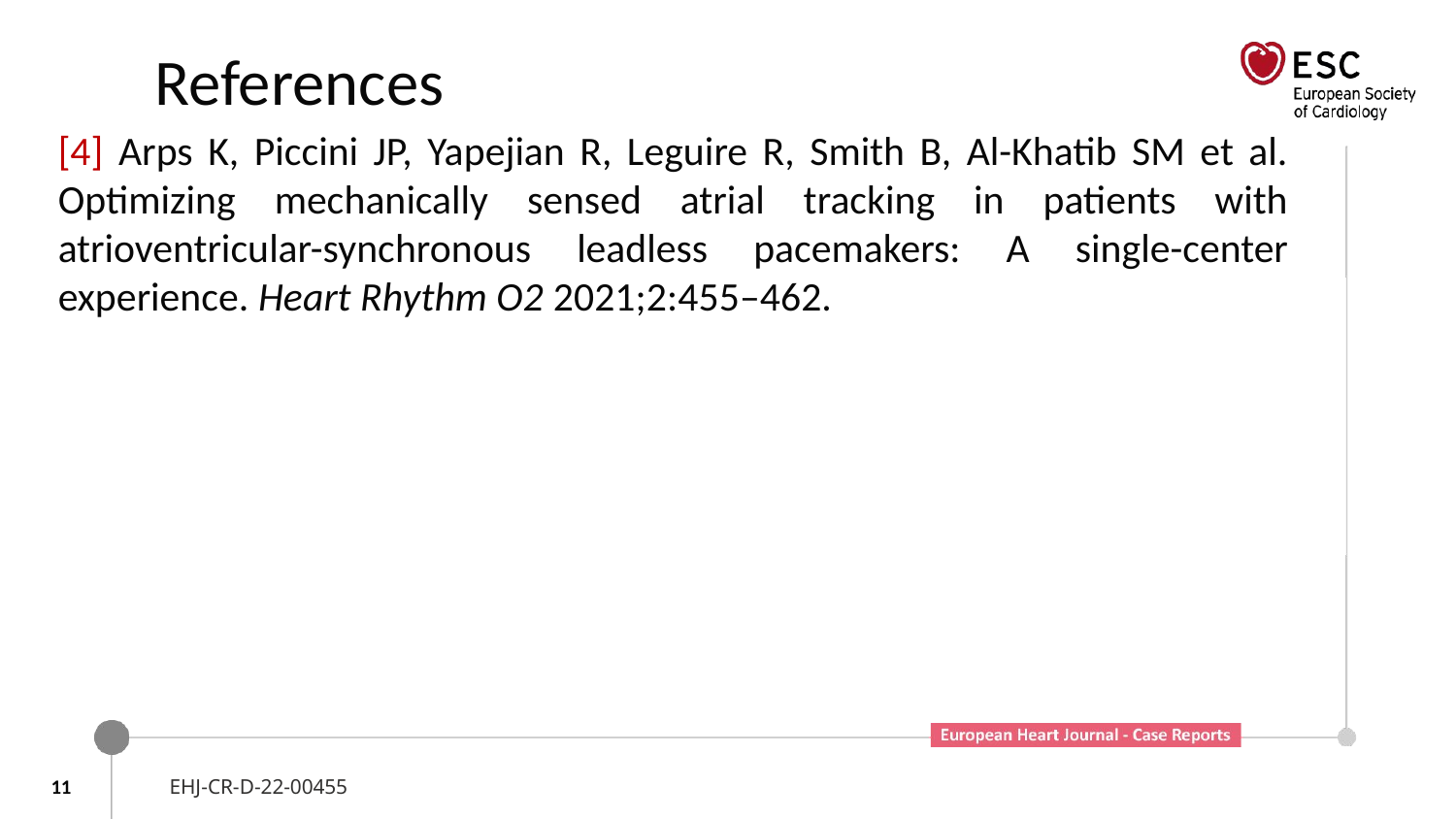

# References
[4] Arps K, Piccini JP, Yapejian R, Leguire R, Smith B, Al-Khatib SM et al. Optimizing mechanically sensed atrial tracking in patients with atrioventricular-synchronous leadless pacemakers: A single-center experience. Heart Rhythm O2 2021;2:455–462.
11
EHJ-CR-D-22-00455
